# Genome Sequencing of Efficient Plant Growth-Promoting Bacteria for Lowland Rice

**DOI:** 10.1128/mra.00185-23

**Published:** 2023-05-23

**Authors:** Milena Serenato Klepa, Maria Laura Turino Mattos, Renan Augusto Ribeiro, Luisa Caroline Ferraz Helene, Marco Antonio Nogueira, Mariangela Hungria

**Affiliations:** a Embrapa Soja, Soil Biotechnology Laboratory, Londrina, Paraná, Brazil; b CNPq, SHIS QI 1 Conjunto B, Lago Sul, Brasília, Federal District, Brazil; c Embrapa Clima Temperado, Pelotas, Rio Grande do Sul, Brazil; Indiana University, Bloomington

## Abstract

The genomes of five elite strains identified as growth promoters of lowland rice (Oryza sativa L.) in Brazil were sequenced. They ranged in size from 3,695,387 bp to 5,682,101 bp, encompassing genes of saprophytic ability and stress tolerance. Genome taxonomy enabled their classification as Priestia megaterium, Bacillus altitudinis, and three putative new species of Pseudomonas, *Lysinibacillus*, and *Agrobacterium*.

## ANNOUNCEMENT

Rice (Oryza sativa L.) is an important crop for food security and human nutrition in Brazil and is included in the daily diet of most of the population; the main production comes from the lowlands in the southern region (1). The use of plant growth-promoting bacteria (PGPB) has increased worldwide, aiming to replace or complement chemical fertilizers (2). A strain selection program was established at Embrapa Temperate Climate (31°52′00″S; 52°21′24″W), focusing on the isolation of strains from rice grown in the lowlands. Samples of rice stems were surface disinfected and crushed in 90 mL of 0.05 M phosphate buffer, and the supernatant was inoculated in N-free broth, resulting in 36 isolates that were evaluated for PGP properties (3). Five promising strains isolated from stacks of harvested rice were evaluated in field experiments for four consecutive years and showed outstanding performance, increasing rice grain yield with a reduction of 90 kg of N-fertilizer per hectare (4). Using these elite strains as commercial microbial inoculants requires genome sequencing.

The strains were grown in tryptic soy broth (TSB) at 28°C for 24 to 48 h, and then total DNA was extracted using the DNeasy blood and tissue kit (Qiagen). Libraries were constructed using the Nextera XT kit, and the genomes were sequenced on the NextSeq 1000 instrument (Illumina) at Embrapa Soybean (Londrina, Brazil). The paired-end reads were assembled using the A5 MiSeq pipeline version 20140604 (5) with default quality control parameters; sequence adapters and low-quality regions were trimmed using Trimmomatic (6), errors were corrected using SGA’s k-mer-based algorithm (7), and the genome was annotated using the NCBI Prokaryotic Genome Annotation Pipeline (PGAP) version 6.4 (8).

The genome sizes ranged from 3,695,387 to 5,682,101 bp, with coverages of at least 255-fold. Between 3,706 and 5,811 coding sequences enriched in genes involved in iron, nitrogen, sulfur, phosphorus, amino acid, vitamin, and cofactor metabolism and several genes related to stress response were identified in all genomes in the PGAP analysis. These genes are indicative of saprophytic ability and tolerance to environmental stresses. More information about the assemblies is provided in Table 1.

**TABLE 1:** Genomic parameters of strains CNPSo 3701 (=CMM 174), CNPSo 3703 (=CMM 176), CNPSo 3705 (=CMM 178), CNPSo 3706 (=CMM 179), and CNPSo 3708 (=CMM 194)

Statistic	Data for strain:				
	Pseudomonas sp. CNPSo 3701	Bacillus altitudinis CNPSo 3703	*Lysinibacillus* sp. CNPSo 3705	Priestia megaterium CNPSo 3706	*Agrobacterium* sp. CNPSo 3708
GenBank accession no.	JAQZSI000000000	JARBGV000000000	JAQZSD000000000	JAQZSE000000000	JAQZSF000000000
SRA accession no.	SRR23468783	SRR23489148	SRR23456433	SRR23456434	SRR23456515
Size (bp)	5,007,775	3,695,387	5,164,077	5,682,101	4,776,167
No. of contigs	39	21	251	131	32
*N*_50_ (bp)	650,953	667,480	65,834	532,791	408,322
Coverage (×)	258	399	305	255	318
G+C content (%)	63.1	41.3	36.6	37.7	58.2
Read length (bp)	100	300	100	100	100
Total no. of reads	13,178,706	5,906,618	19,567,840	15,279,238	16,570,560
No. of coding sequences	4,593	3,706	4,945	5,811	4,411
No. of RNAs	64	115	106	143	58
Closest species (GenBank accession no. or JGI GOLD project ID)	Pseudomonas straminea JCM 2783^T^ (GCF_900112645.1)	Bacillus altitudinis 41KF2b^T^ (GCF_000691145.1), Bacillus aerius 24K^T^ (Gp0506539)	Lysinibacillus xylanilyticus DSM 23493^T^ (GCF_001183605.1)	Priestia megaterium ATCC 14581^T^ (GCF_017086525.1)	Agrobacterium cavarae RZME10^T^ (GCF_004310285.1)
ANI^*a*^ (%)	96.13	98.10, 98.74	91.27	97.41	96.24
dDDH^*b*^ (%)	67.5	83.3, 89.1	45.2	77.0	68.6

a ANI, average nucleotide identity (calculated using OrthoANIu).

b dDDH, digital DNA-DNA hybridization.

Complete 16S rRNA gene sequence comparisons were carried out with species of each genus to select the closest species, defined as sharing ≥98.7% similarity (9). The average nucleotide identity (ANI) (10) and digital DNA-DNA hybridization (dDDH) (11) were calculated with the closest type strains. Strain CNPSo 3706 was classified as Priestia megaterium. Strain CNPSo 3703 shared ANI and dDDH values above 98% and 83%, respectively, with two *Bacillus* species; however, as Bacillus aerius is no longer recognized as a species (12), strain CNPSo 3703 was classified as Bacillus altitudinis. The other three strains are putative new species belonging to the genera Pseudomonas, *Lysinibacillus*, and *Agrobacterium* (Table 1).

These results highlight the high diversity of plant growth-promoting elite bacterial strains identified in the Brazilian lowlands cropped to rice. Proper genomic identification will allow their use as microbial inoculants for rice, bringing economic and environmental benefits.

### Data availability.

The genomic data and reads were deposited at DDBJ/ENA/GenBank under BioProject accession number PRJNA933527. The BioSample, GenBank, and Sequence Read Archive accession numbers are, respectively, SAMN33238823, JAQZSI000000000, and SRR23468783 for CNPSo 3701; SAMN33271202, JARBGV000000000, and SRR23489148 for CNPSo 3703; SAMN33271204, JAQZSD000000000, and SRR23456433 for CNPSo 3705; SAMN33271201, JAQZSE000000000, and SRR23456434 for CNPSo 3706; and SAMN33271203, JAQZSF000000000, and SRR23456515 for CNPSo 3708.
